# Comparison of strategies for scalable causal discovery of latent variable models from mixed data

**DOI:** 10.1007/s41060-018-0104-3

**Published:** 2018-02-06

**Authors:** Vineet K. Raghu, Joseph D. Ramsey, Alison Morris, Dimitrios V. Manatakis, Peter Sprites, Panos K. Chrysanthis, Clark Glymour, Panayiotis V. Benos

**Affiliations:** 1 Department of Computer Science, University of Pittsburgh, Pittsburgh, PA USA; 2 Department of Computational and Systems Biology, University of Pittsburgh, Pittsburgh, PA USA; 3 Department of Philosophy, Carnegie Mellon University, Pittsburgh, PA USA; 4 UPMC Department of Medicine, Pittsburgh, PA USA

**Keywords:** Causal inference, Latent variables, Mixed data, COPD, HIV

## Abstract

Modern technologies allow large, complex biomedical datasets to be collected from patient cohorts. These datasets are comprised of both continuous and categorical data (“Mixed Data”), and essential variables may be unobserved in this data due to the complex nature of biomedical phenomena. Causal inference algorithms can identify important relationships from biomedical data; however, handling the challenges of causal inference over mixed data with unmeasured confounders in a scalable way is still an open problem. Despite recent advances into causal discovery strategies that could potentially handle these challenges; individually, no study currently exists that comprehensively compares these approaches in this setting. In this paper, we present a comparative study that addresses this problem by comparing the accuracy and efficiency of different strategies in large, mixed datasets with latent confounders. We experiment with two extensions of the Fast Causal Inference algorithm: a maximum probability search procedure we recently developed to identify causal orientations more accurately, and a strategy which quickly eliminates unlikely adjacencies in order to achieve scalability to high-dimensional data. We demonstrate that these methods significantly outperform the state of the art in the field by achieving both accurate edge orientations and tractable running time in simulation experiments on datasets with up to 500 variables. Finally, we demonstrate the usability of the best performing approach on real data by applying it to a biomedical dataset of HIV-infected individuals.

**Electronic supplementary material:**

The online version of this article (10.1007/s41060-018-0104-3) contains supplementary material, which is available to authorized users.

## Introduction

Directed graphical causal models are an indispensable tool for predicting the outcomes of interventions on variables from purely observational datasets in domains ranging from economic to biological data. Biological datasets in particular present a challenge in that they typically include both categorical variables and continuous variables (“Mixed Data”) and that a number of causal variables may be unknown or impossible to measure. Though research in graphical causal models has been ongoing for several years [1, 9, 15, 21, 26] most algorithms on mixed data have been developed to learn only undirected graphs (not causal). Tur and Castelo [29] address the problem of learning mixed graphical models with very low sample size relative to the number of variables, under the assumption of a Conditional Gaussian (CG) model built upon the framework of [11]. In [2], the authors proposed a simplification of the CG model using a group lasso type sparsity penalty to work with high-dimensional data. Both [13] and [7] study special cases of the CG model that significantly reduce the number of free parameters, enabling scalability to larger datasets. We chose to focus upon the model from [13] due to its efficiency and its success in prior applications to biological data [23]. Though structure learning algorithms for mixed data have focused on the undirected case, causal models can be learned on mixed data using constraint-based causal discovery algorithms such as the PC [26] algorithm, given that an independence test suitable for mixed variables is used.

One important assumption that many causal modeling algorithms rely upon is the causal sufficiency assumption [26], which states that there are no unmeasured variables (latent confounders) causally affecting at least two observed variables in the system, but in practice, it is unrealistic to assume that this is the case for any biological system. Another important factor in developing these algorithms is that the sizes of many biological datasets have expanded rapidly in the past years as a result of new technologies for high-throughput data collection. Thus, in order for causal inference algorithms to be practical, they need to be able to process big data efficiently [28].

The standard constraint-based causal discovery algorithm for latent variables has been the Fast Causal Inference (FCI) algorithm [27]. However, the word ”fast” in the name of the algorithm was meant to signify its superior speed compared to the state of the art at that time, and for high-dimensional data the algorithm is not particularly fast nor highly accurate. There have been a number of proposed extensions to improve the speed and output accuracy of the algorithm. Really Fast Causal Inference (RFCI) is a modification of FCI that uses fewer independence tests than FCI to achieve a significant speedup at the cost of reduced information in the final output graph [5]. Anytime FCI is a modification of FCI that allows the algorithm to terminate FCI’s original conditional independence search early with the guarantee that no incorrect information will be presented, though the output may again be less informative than a full run of FCI [25].

Several other algorithms have been proposed to handle the presence of latent variables in causal discovery. The FindOneFactorClusters (FOFC) algorithm imposes restrictions on the graphical structure of the true causal model, thereby rendering it unsuitable to the general causal discovery problem [10]. Previously Overcomplete ICA was used to learn a causal ordering among the variables which allows an experimenter to learn experimental predictions; however, this approach is limited in scale and requires few latent variables relative to sample size [8]. In addition, none of these approaches are intended to deal with both continuous and categorical data simultaneously.

GFCI [14] is a new procedure that uses a parallelization of the Greedy Equivalence Search algorithm (GES) [18] as an initial step before running FCI; however, to apply this algorithm to mixed variables requires a hybrid scoring function which has not yet been formulated. Sokolova et al. [24] have developed a scoring function for mixed data that has unsuitable assumptions for categorical data. Their original algorithm, the Bayesian Constraint-Based Causal Discovery algorithm (BCCD) [3] uses a hybrid constraint and score-based approach to perform causal search in the presence of latent variables. The extension of this work to handle both discrete and continuous variables uses a modification of the Bayesian Information Criterion (BIC) score. However, this mixed score assumes monotonic relationships between the variables, and is only computationally feasible for relatively small datasets.

FCI-Stable is an adaptation of FCI that resolves the order-dependency of the algorithm and allows for straightforward parallelization [4]. This approach is now commonly used in place of the FCI algorithm, and thus we chose to use FCI-Stable as a baseline in this paper because this algorithm is more robust than the original FCI and can accommodate a suitable independence test for mixed data. In addition, we include the BCCD approach in our experiments, since it can handle mixed datasets, albeit with less scalability and with slightly restrictive assumptions.

We present a comparison of strategies for causal discovery in the presence of both latent variables and mixed data. One strategy utilizes a maximum probability-based search technique that was first applied to the PC algorithm [16]. In preliminary studies, this method, entitled PC-Max, performed well in reference to the state of the art in causal discovery without latent variables. The approach examined here, called FCI-MAX, uses a similar max search technique in the edge orientation portion of FCI to determine which conditioning sets of variables are most likely to provide correct conditional independence facts. It also utilizes the foundation of FCI to extend PC-Max to the latent variable case. Though this maximum probability procedure improves the accuracy of the causal orientations of the FCI algorithm, it requires significantly more independence tests, thereby reducing the scalability of the algorithm even further. To combat this, we employed a preprocessing technique suitable for mixed data types called Mixed Graphical Models (MGM) [23], an improvement over [13], to quickly eliminate unlikely edges in our output causal graph. MGM helps learn the correct undirected skeleton before orienting edges using our new conditional independence tests with FCI.

Our major contributions are as follows:

- We present and compare *four new strategies* to learn causal graphs over mixed data types with latent variables: FCI-MAX, MGM-FCI-MAX, MGM-FCI, and MGM-CFCI (Sect. 3).
- We provide optimizations and parallelization of the FCI-MAX algorithm to further improve performance (Sect. 3.3).
- We present an extensive experimental evaluation of these algorithms on a variety of simulated datasets against the baseline FCI algorithm and against BCCD (Sects. 4 and 5)
- We demonstrate an application of our approach to a real biological dataset with latent confounders (Sect. 5.3).

## Background and assumptions

Much of the terminology and assumptions of this work stem from those mentioned in [26], and we refer the reader to that work and Online Resource 1 for a detailed description. Here, we describe the original state of the art algorithm for causal discovery in the presence of latent variables: Fast Causal Inference (FCI) and the output of the algorithm, a Partial Ancestral Graph (PAG).

### Fast Causal Inference

We originally developed FCI, which learns a causal PAG from data that may include latent variables [27]. The algorithm proceeds by using conditional independence tests akin to our PC algorithm [26] to quickly uncover an undirected skeleton of the true output graph, and subsequently colliders are oriented in the same manner as the original PC algorithm. In order to achieve correctness in the causally insufficient case (the case when latent variables are present), the algorithm must then further test some conditioning sets among non-adjacent variables. This is due to the fact that when the causal sufficiency assumption holds, if two variables are conditionally independent then they are conditionally independent given some subset of their adjacencies. However, in the causally insufficient case this no longer holds. This phase is referred to as the Possible D-Sep phase, as the variables that may be in a separating set for a given pair of variables is characterized as the Possible D-Sep set. Finally, all edges are reoriented to have both circle endpoints, and then orientation rules are applied as given by Zhang [30].

For robustness, we use the stable version of this algorithm (FCI-Stable). This version alters the undirected skeleton search at the onset of the algorithm to erase the order-dependency of the FCI algorithm. This is done by deleting edges only after all independence tests at the current conditioning set size have been performed. Henceforth, for simplicity we use the label "FCI" to mean the FCI-Stable algorithm.

Conservative FCI (CFCI) [17] is a modification of FCI that assumes a weaker version of faithfulness called adjacency-faithfulness. The algorithm performs extra independence tests for each potential unshielded collider (A–B–C) to determine whether or not the variable B appears in all sets that separate A and C or none of the sets that separate A and C. In the first case, the algorithm orients as a non-collider, whereas in the second case the algorithm orients as a collider. If B appears in some separating sets but not others, the algorithms marks the collider as unfaithful and determines that no information can be extracted from the data. In practice, CFCI tends to give more reliable output at the expense of predicted colliders.

### Partial Ancestral Graph

A PAG is the representation of the output of the FCI algorithm, and it is a causal graph suitable for situations in which latent variables may be present. This type of graph consists of three different types of endpoints: (o , >, −), and each edge here represents ancestral relationships between the nodes of the graph. $\hbox {A} \rightarrow \hbox {B}$ denotes that A is an ancestor of B, and $\hbox {A} \leftrightarrow \hbox {B}$ denotes that there is a latent variable causing both A and B. The circular endpoint denotes uncertainty about the correct causal endpoint, e.g., A o$\rightarrow$ B could be $\hbox {A} \rightarrow \hbox {B}$ or $\hbox {A} \leftrightarrow \hbox {B}$ in the true graph, and thus the only certain knowledge from this orientation is that B is not an ancestor of A. We again refer the reader to [26] for a thorough theoretical grounding in these concepts.

## Methods

### Fast Causal Inference-Max (FCI-MAX)

Fast Causal Inference-Max (FCI-MAX) is a new modification of FCI that improves causal orientations. During the first collider orientation phase of FCI, the algorithm orients an unshielded collider (X—Y—Z) by determining whether Y does not appear in the conditioning set that renders X and Z conditionally independent (defined as the ”separating set” of X and Z). The FCI algorithm does this by simply using the first separating set that it finds during the adjacency search as the true separating set of X and Z. On the other hand, FCI-MAX performs a conditional independence test on all possible subsets of the adjacencies of X and Z and uses the one with the largest p value in its conditional independence test as the true separating set. The intuition for this method is that the conditioning set with the largest *p* value represents the set least likely to imply dependence since likelihood for dependence falls off monotonically with increasing *p* value. [16] Next, the algorithm runs the Possible D-Sep stage as normal, and uses the max technique again in order to orient unshielded colliders following this stage. Finally, the other orientation rules are applied as usually done in FCI.

### Independence test for mixed data

For both of the Fast Causal Inference algorithms, a reliable conditional independence test is necessary for proper output. For the mixed data in this study, we had developed a new conditional independence test based upon linear and logistic regressions [22]. First, all discrete variables are transformed into several binary indicator variables, one for each category of the original discrete variable. Then, to test the independence of two continuous variables *X* and *Y*, given a conditioning set *S*, we perform a linear regression of *X* onto *Y* and *S*, and we perform a *t* test on the coefficient of *Y* in this regression. If the *p* value of this test is less than a specified threshold $\alpha$, we reject the null hypothesis of zero partial correlation between *X* and *Y* and determine that *X* and *Y* are conditionally dependent given *S*. Note that in this situation *S* may contain both continuous and categorical variables and for the categorical variables, the aforementioned binary indicator variables are used as the predictors. Alternatively, if *X* or *Y* is a categorical variable, then we utilize a likelihood ratio test between logistic regression models including the conditioning set *S* as predictors (null model) and without the conditioning set as predictors.

### Parallelization and optimization

In the past, causal learning algorithms have not scaled well to the challenges of Big Data, with few exceptions [18]. In this section, we discuss parallelization and optimizations for FCI-MAX to render a more realistic runtime on high-dimensional data. Since FCI-based algorithms use the PC adjacency search to find an undirected skeleton, this search was implemented in parallel using the technique given by [12] without modification. Unfortunately, parallelizing this portion alone is not enough to even equal the runtime of the FCI algorithm due to the extra conditional independence tests necessary to find the maximum *p* value conditioning set. Thus, for FCI-MAX, we parallelize the collider orientation phase as well, by utilizing a scheme in which each thread was given a job requiring a similar amount of independence tests, computed based upon the number of edges adjacent to the nodes involved in the current collider.

In particular, the task to orient all colliders was subdivided recursively and split into two jobs if the total number of adjacent edges among all of the involved colliders in the current job was greater than the chunk size, which was set to the total number of edges in the graph $(\vert E \vert )$ multiplied by $\beta$ (a user-specified parameter) divided by the number of available processors $(\vert \textit{Cores}\vert )$. Let $\textit{Adjacent}(X,G)$ be the set of nodes adjacent to node *X* in graph G. Then, given *n* unshielded colliders $(X_{i} - Y_{i} - Z_{i})$, and Graph $G = (V,E)$ the following rule defines when recursive subdivisions occur:

1 $$\begin{aligned} \begin{aligned}&\text {If}\, \bigg (\bigg (\sum \limits _{i}\vert {\textit{Adjacent}}(X_{i},G)\vert + \vert {\textit{Adjacent}}(Z_{i},G)\vert \bigg ) \\&\quad > \frac{\vert E \vert }{\vert Cores \vert }\bigg ) \, {\textit{then}} \, {\textit{Subdivide}} \end{aligned} \end{aligned}$$

The $\textit{Subdivide}$ operation splits a job (which would normally be assigned to a single thread) into two jobs which can be executed in parallel by multiple threads. If these jobs are still too large by the above rule, then they can be further subdivided, etc. In addition, an optimization to the FCI-MAX algorithm was included involving the Possible D-Sep phase. Often times, this phase results in no change to many adjacencies of the graph. Taking advantage of this, we retain colliders whose adjacency sets were unchanged by the Possible D-Sep phase instead of retesting them to determine orientations. This change results in no loss of accuracy and often times can lead to a substantial reduction in runtime by eliminating redundant independence tests. Note that some independence tests will still be repeated in our current scheme since caching all independence test results is exponential in the number of nodes in the graph. It is future work to explore this time-memory tradeoff to determine if caching more independence tests is beneficial for FCI-MAX.

### Mixed Graphical Models

A Mixed Graphical Model (MGM) is an undirected graphical model proposed by Lee and Hastie [13] to characterize the joint distribution over a dataset with both continuous and discrete variables, and it is given by the following expression:

2 $$\begin{aligned}&p(x,y;\theta ) \propto \mathrm{exp}\bigg (\sum \limits _{s=1}^{p}\sum \limits _{t=1}^{p} -\frac{1}{2}\beta _{st}x_{s}x_{t} \nonumber \\&\quad +\sum \limits _{s=1}^{p}\alpha _{s}x_{s} + \sum \limits _{s=1}^{p}\sum \limits _{j=1}^{q}\rho _{sj}(y_{j})x_{s} + \sum \limits _{j=1}^{q}\sum \limits _{r=1}^{q}\phi _{rj}(y_{r},y_{j})\bigg )\nonumber \\ \end{aligned}$$

where $\theta$ represents the full set of parameters, $x_{s}$ represents the $s\mathrm{th}$ of *p* continuous variables and $y_{j}$ represents the $j\mathrm{th}$ of *q* discrete variables. $\beta _{st}$ represents the potential for an edge between continuous variables *s* and *t*, $\alpha _{s}$ represents the potential for a node of a continuous variable, $\rho _{sj}$ represents the potential for an edge between continuous variable *s* and discrete variable *j*, and finally $\phi _{rj}$ represents the potential for an edge between discrete variables *r* and *j*. This model has the favorable property that its conditional distributions are given by Gaussian linear regression and Multiclass Logistic Regression for continuous and discrete variables, respectively.

3 $$\begin{aligned} \begin{aligned} \widetilde{l}({\varTheta }\vert x,y) =&-\sum \limits _{s=1}^{p} \log {p(x_{s}\vert x_{\slash s}, y; {\varTheta })}\\&- \sum \limits _{r=1}^{q}\log {p(y_{r}\vert x,y_{\slash r}; {\varTheta })} \end{aligned} \end{aligned}$$

With this characterization, one can find conditional independence relationships between each pair of variables given the rest of the variables in the graph, which in turn provide an undirected graphical structure. Learning this model over high-dimensional datasets directly is computationally infeasible due to the computation of the partition function, so to avoid this, a proximal gradient method is used to learn a penalized negative log pseudolikelihood form of the model. This negative log pseudolikelihood is given in Eq. 3, and the penalized form is presented in Eq. 4 and described in [23]. For both of these, ${\varTheta }$ refers to all parameters of the model collectively.

4 $$\begin{aligned} \begin{aligned} \underset{{\varTheta }}{\text {minimize}} \, l_{\lambda }({\varTheta })&= \widetilde{l}({\varTheta }) + \lambda _{\textit{CC}} \sum \limits _{s=1}^{p}\sum \limits _{t=1}^{s-1}\vert \beta _{st}\vert \\&\quad + \lambda _{\textit{CD}} \sum \limits _{s=1}^{p}\sum \limits _{j=1}^{q} \vert \vert \rho _{sj}\vert \vert _{2} \\&\quad + \lambda _{\textit{DD}}\sum \limits _{j=1}^{q}\sum \limits _{r=1}^{j-1}\vert \vert \phi _{rj}\vert \vert _{F} \end{aligned} \end{aligned}$$

We use the algorithm as specified in [23] with the modification for efficiency that instead of waiting for the edge parameters themselves to converge, we terminate the algorithm when the number of different edges in the graph produced by the algorithm remains unchanged for three consecutive iterations. For the hybrid algorithms proposed in this paper, we use the Mixed Graphical Model (MGM), as a constraining input graph to the other algorithms (instead of beginning with the fully connected graph as FCI typically does). Thus, the parameters themselves are not important for the remainder of these algorithms, as only the undirected graphical structure is used.

We finally note that using the MGM approach to learn an undirected graph does not affect the theoretical correctness guarantee of the FCI algorithm. This correctness guarantee will persist as long as MGM learns a *superset* of the adjacencies learned in the first phase (skeleton identification) of FCI. In the sample limit, without confounding variables, MGM will learn a ’moralized graph,’ which is a graph consisting of the true adjacencies in the data generating graph, along with an edge between all pairs of nodes that have a common child. We note that including confounding variables can only increase the edges found between observed variables, as confounding can only induce spurious edges, and not remove existing edges. Thus, since the undirected graph learned by MGM consists of the true edges, edges caused by moralization, and spurious edges from latent confounding, it must be a superset of the true adjacencies. Since the undirected graph is a superset of the true edges, FCI’s theoretical correctness still applies to give overall correctness for the algorithm.

**Table 1:** Experimental orientation score

	Predicted: A *-o B	Predicted: A *-> B	Predicted: A *– B
True edge: A *-o B	1 TP	If($\sim$Ancestor(B,A)) 1 TP	If(Ancestor(B,A)) 1 TP
		Else, 1 FN	Else, 1 FP
True edge: A *-> B	$\frac{1}{2}$ FP	1 TP	1 FP
True edge: A *– B	$\frac{1}{2}$ FN	1 FN	1 TP

## Experimental evaluation

In our evaluation, seven algorithms were tested on simulated datasets. These included the baseline FCI-Stable algorithm (referred to as FCI), FCI-MAX, CFCI, FCI with the MGM skeleton (MGM-FCI), FCI-MAX with the MGM skeleton (MGM-FCI-MAX or MFM), CFCI with the MGM skeleton (MGM-CFCI) and the Bayesian Constraint-Based Causal Discovery Algorithm (BCCD).

### Evaluation metrics

The algorithms were evaluated based on each type of edge present in the graph: Edges between two continuous variables (CC), between a continuous and a discrete variable (CD), and between two discrete variables (DD). For each of these edge types the output PAG of the algorithms was compared to the true PAG that FCI would identify if it had a conditional independence oracle. Intuitively, this PAG is the best possible result that could come from using correct conditional independence information alone. The true PAG and the output PAG were compared in order to compute adjacency precision, adjacency recall, orientation precision, and orientation recall. Precision and recall refer to the standard definitions used in the machine learning literature, where a true positive adjacency refers to an edge that appears in both the estimated PAG and the true PAG regardless of its orientation, a false positive adjacency is an edge that appears only in the estimated PAG, and a false negative adjacency refers to an edge that only appears in the true PAG.

Orientation precision and recall are computed using the true positive, false positive, and false negative information in Table 1. This score is an attempt to quantify the amount of useful information a biologist would gain from examining the output PAG in terms of suggesting ancestral information between variables (as this is the information depicted in a PAG). This score utilizes a precision and recall measurement instead of a single numerical value. Note that this score is only computed on edges that appeared in both the true PAG and the estimated PAG. Thus when this score is presented it will always be accompanied by an adjacency score to separate the information gained from the adjacencies found by our algorithms versus the orientations found by our algorithms.

The main idea of the score is to award a true positive if the estimated PAG matches the output PAG; however, unlike a method such as Structural Hamming Distance, we examine the actual predicted endpoint to determine causal meaning. In particular, for an edge A *-x B (where * is a wildcard for any endpoint), replacing “x” with the endpoint “>” is an implication of non-ancestry from B to A (either because A causes B or because they are mediated by a latent variable). On the other hand, replacing “x” with the endpoint “-” in the absence of selection bias is an implication of ancestry from B to A. Thus, in our score a false positive refers to an incorrect prediction of ancestry and a false negative is an incorrect prediction of non-ancestry. When the ground truth PAG has a varying endpoint (“o”), the data generating DAG is consulted to determine ancestral relationships. When the predicted endpoint is “o” this is given a partially incorrect score unless the endpoint in the true PAG is also "o". In Table 1, Ancestor(A,B) is true if A is an ancestor of B in the data generating DAG, and $\sim$ refers to a boolean NOT operation.

### Datasets

#### Synthetic data

Our methods were tested on random graphs consisting of half continuous and half discrete variables. The number of nodes in these graphs included 50 and 500 variables. The discrete variables had three categories, and the graphs had varying edge densities. The 50 node networks had edge amounts normally distributed with a mean of 100 edges and a standard deviation of 30, while the 500 node networks had edge amounts normally distributed with a mean of 750 edges and a standard deviation of 200. The full statistics for all simulated graphs are given in Online Resource 1.

In order to produce a network with the potential for latent confounders each network had 5 or 20 variables selected to act as latent variables for the 50 and 500 node networks, respectively. Thus, these nodes were removed from the dataset used for the algorithmic search procedures. To ensure that these variables truly created confounding effects, only variables with at least two observed children were selected to become ”latent”. To convert this original DAG with latent variables to a Partial Ancestral Graph (PAG), an oracle for conditional independence relations from the underlying DAG along with the set of nodes from the DAG (excluding the latent variables) are given as input to the FCI algorithm, and the output is set as the true PAG for comparison [19].

Datasets were generated from the underlying network using a method similar to the one proposed in [13]. Parents contribute to the values of their children in different ways depending upon the type of parent and the type of child. Parents of continuous variables contribute linearly to the means of their children. In particular, continuous parents are multiplied by a single edge parameter, whereas discrete parents have a distinct edge parameter associated with each category of the discrete variable. Parents of discrete variables contribute log-linearly to the probabilities of each category of their children, with separate edge parameters for each category of the child. Thus, when discrete variables have 3 categories, edges connecting two continuous variables consist of a single edge parameter, edges connecting a continuous and discrete variable consist of 3 parameters, and edges connecting two discrete variables consist of 9 parameters.

**Fig. 1:**
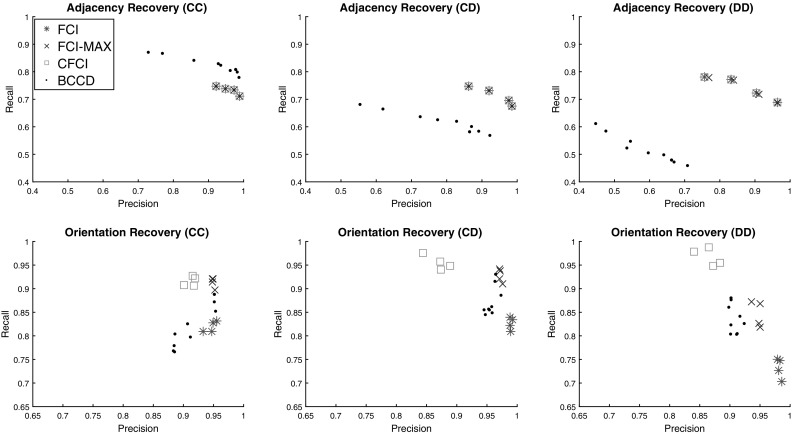
Precision-recall plots of FCI and related algorithms for 50 node scale-free networks with 1000 samples and 5 latent variables. Plots are separated by adjacency and orientation recovery in each row, and by edge type in each column; (CC), (CD) and (DD) refer to edges between two continuous variables, continuous and discrete variables, and two discrete variables, respectively

Edge weights were drawn uniformly at random from the union of the regions $\left[ -1.5,.5\right]$ and $\left[ .5,1.5\right]$. For continuous-continuous (CC) edges, the edge parameter is equal to the drawn weight. For continuous-discrete (CD) edges, a vector of 3 values are drawn uniformly from $\left[ 0,1\right]$, and these values are scaled such that they sum to 0 and shifted so that the largest edge parameter is equal to the original drawn weight. For discrete-discrete (DD) edges, we use the same method as for CD edges, except that we use three permutations of the original vector to fill all 3 rows of the matrix. To ensure the discrete variables behaved as categorical variables, the CD and DD parameters were permuted such that no edge relationship between two variables was monotonic. In this manner, treating all relationships as linear would not suffice in producing correct output, as would be the case with categorical variables in practice.

With these parameters, data were generated using structural equation models from the network. For discrete variables, we first make a random draw uniformly from the interval $\left[ 0,1\right]$ to be used as an error term that determines the actual value of the variable given the probabilities of each category. For continuous variables, Gaussian error terms with mean equal to zero, and standard deviation uniformly drawn from $\left[ 1,2\right]$ are set for each variable. Since the dataset is based off of a DAG structure, convergence of the mean values for the variables occurs by starting at the root nodes of the graph, and propagating the values of children downwards from this starting point. To simulate latent variables in our dataset, we take our original simulated dataset and remove variables corresponding to the variables set as latent in the true PAG.

#### Lung human immunodeficiency virus (HIV) dataset

This real dataset consisted of a mixture of categorical and continuous variables consisting of indicators of lung function status, standard demographic information, smoking and medication history for 933 individuals. These individuals were both HIV positive and HIV negative with varying levels of lung function decline. To deal with missing data in this set, individuals (samples) and variables with greater than 10% missing values were removed. In addition, variables with variance smaller than .01 were removed. The overall goal in analyzing this dataset was to identify interesting causal relationships and to validate our method by identifying known biological associations between variables for just the HIV positive individuals. Thus, our final dataset consisted of 29 categorical variables and 14 continuous variables with 424 samples.

**Fig. 2:**
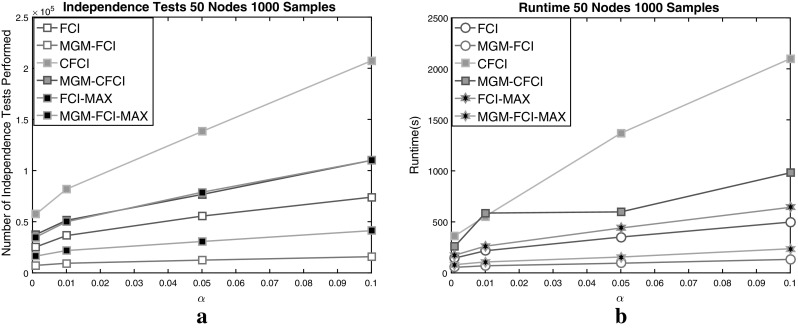
Runtime experiment with 50 nodes, 1000 samples, and 5 latent variables. All MGM algorithms were set to $\lambda = .15$. **a** Number of independence tests performed, **b** wall clock time

## Results

Here we discuss the major findings of the paper. We begin by examining both the accuracy and runtime of the algorithms using the smaller 50 node synthetic datasets. We then discover how well these algorithms scale to 500 node datasets, and we finally discuss the effectiveness of the best performing approach on the HIV biological dataset.

### 50 node results

To discover the contributions of the various strategies to improve the FCI algorithm, we first compare FCI with its variants (FCI-MAX and CFCI) along with BCCD to determine the most accurate and efficient algorithms. Then we incorporate the MGM strategy to the best performing algorithms to achieve scalability while maintaining accuracy.

#### FCI and variants

Our first experiment aimed to determine which algorithms were most accurate and efficient on 50 node datasets. Figure 1 shows accuracy results for these algorithms across both Adjacency Recovery and Orientation Recovery for each edge type: continuous-continuous (CC), continuous-discrete (CD), and discrete-discrete (DD). In these plots, a single data point refers to an average precision and recall measurement for a particular setting of parameters across all the graphs. For each trial, $\alpha$ was selected from the set $\left[ .001, .01, .05, .1\right]$ to determine the impact of the parameter across a range of values. For BCCD, we used all combinations of edge probability cutoffs and orientation probability cutoffs chosen from the set $\left[ .25, .5, .75\right]$, and so there are nine data points for this algorithm. The three columns of the figure correspond to edges between different types of variables (CC, CD, DD).

All FCI modifications produced similar adjacencies but the adjacency recovery for all these algorithms is significantly better than BCCD for edges involving categorical variables (CD, DD) and slightly worse for CC edges. This is expected since BCCD can handle monotonic relationships well (which applies to the CC edges only). In addition, we see that among the correctly identified adjacencies, FCI-MAX tends to orient these with the best balance between precision and recall, whereas CFCI has high recall in all cases, and FCI has high precision in all cases. This is due to the fact that CFCI refrains from orienting many edges with arrowheads (thereby avoiding false negatives), and FCI orients nearly all edges as arrowheads (thereby avoiding false positives). We believe this to be because FCI uses the first separating set it finds as the true separating set, which results in inaccurate orientations on high-dimensional data. Both of these scenarios are not ideal, as it becomes difficult to discern true causal relationships for both CFCI and FCI. In addition, we note that FCI-MAX tends to outperform BCCD in all cases either by having similar precision and better recall or similar recall and better precision

This experiment was repeated for a more realistic sample size of the data (200 Samples, Online Resource 2) and similar results were obtained. The major difference that we note in this underpowered experiment is the larger spread of the data points across different parameter settings, thereby increasing the importance of selecting a suitable value for the $\alpha$ parameter. A nice feature of these algorithms for adjacencies is that the $\alpha$ value tends to balance between precision and recall (a high value for $\alpha$ leads to good recall and poorer precision, and a low value leads to the opposite).

#### Incorporating MGM for fast skeleton discovery

Though the FCI modifications (CFCI and FCI-MAX) fair well in accuracy, we next examine the runtime of these algorithms for the same 50 node, 1000 sample synthetic datasets on a four core machine. We display runtime both in terms of wall clock time (Figure 2b) and in terms of the number of independence tests performed (Figure 2a) because wall clock time can be influenced by algorithm independent factors (CPU Specs, Sever load, etc.). BCCD was not included in this experiment as the available implementation was in MATLAB, which would result in a biased comparison since all other algorithms were written in Java. In addition, since BCCD is a hybrid constraint and score-based algorithm, measuring the performance by number of independence tests was not a suitable substitute. These figures demonstrate that CFCI performs significantly more independence tests and is thereby significantly slower than the other algorithms. Similarly, FCI-MAX is significantly slower than FCI. Since FCI is known to not scale well to high-dimensional datasets, we note that FCI-MAX and CFCI will also need to have a reduced runtime in order to scale.

**Fig. 3:**
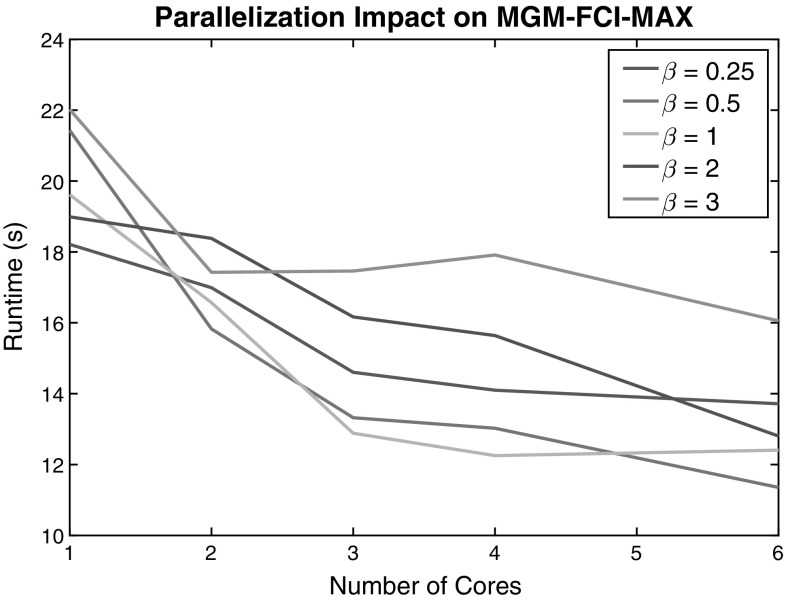
Runtime (s) measured as a function of the number of processors for varying chunk size parameter $\beta$ on 50 node networks with 1000 samples

**Fig. 4:**
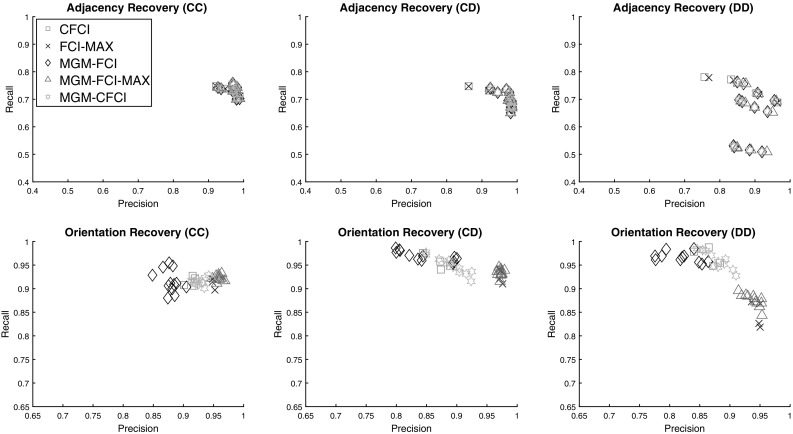
Precision-recall plots with the MGM approach applied to the FCI algorithms for 50 node scale-free networks with 1000 samples and 5 latent variables. FCI-MAX and CFCI are included as a comparison to Fig. 1

However, in the same figure we note the runtime improvements provided by using the MGM approach. For all FCI modifications, MGM improves runtime well above the respective FCI modification. In particular, MGM-FCI and MGM-FCI-MAX improve runtime to faster levels than the FCI baseline approach itself. Clearly, the number of independence tests performed when using the MGM approach is significantly less than the standard approaches, and thus the overhead for computing the MGM graph is the only drawback. Note that these results are presented for a low value of $\lambda = .15$ which actually require the largest runtime, as low values of $\lambda$ tend to produce the densest graphs and thereby require the most independence tests. We demonstrate on the 500 node networks that increasing the value of $\lambda$ can lead to even more significant runtime savings.

**Fig. 5:**
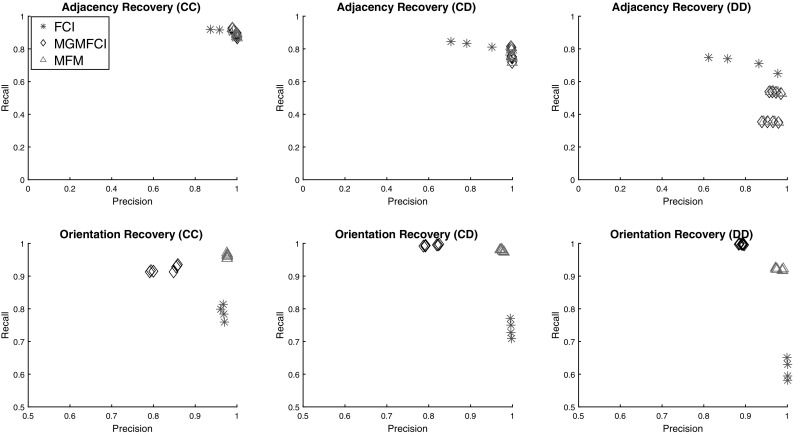
Precision-recall plots of FCI, MGM-FCI, and MGM-FCI-MAX for 500 node scale-free networks with 500 samples and 20 latent variables. Plots are separated by adjacency and orientation recovery in each row, and by edge type in each column; (CC) refers to only edges between two continuous variables, (CD) refers to edges between continuous and discrete variables, while (DD) refers to edges between two discrete variables

The parallelization applied to MGM-FCI-MAX was also examined. Figure 3 shows the effect on runtime of changing the chunk size and the number of cores available for processing. The chunk size determines how small the recursive subdivisions in the orienting colliders phase should be before processing begins. Thus, a larger chunk size means that processing will begin earlier on larger jobs. The figure clearly displays that parallelization has a significant impact on performance, but that the impact has diminishing returns for a larger number of cores. The chunk size factor $\beta$ does not have a straightforward relationship to runtime as the optimal chunk size depends on the number of cores used. However, $\beta$ equal to or less than 1 appears to perform well across all scenarios as long as some parallelism is employed (greater than 1 core).

In Fig. 4, we display the accuracy of results for adding the MGM approach to the FCI modifications with $\lambda$ chosen from the set [.1 .15 .25] for MGM. The best performing FCI modifications without MGM were also included in these graphs for comparison purposes (FCI-MAX and CFCI). We note that MGM does not appear to have a significant impact on the learned causal graphs in most cases. MGM-CFCI marginally improves over the original CFCI algorithm in causal orientations, and MGM-FCI changes the FCI orientations to favor recall instead of precision. MGM-FCI-MAX and FCI-MAX are nearly indistinguishable across most parameter settings for CC and CD edges. We note that some parameter settings for MGM tend to hurt adjacency recall on DD edges. We believe that this is due to the fact that in these experiments, equal $\lambda$ parameter values were used for all types of edges, though it is known that MGM tends to perform better when a smaller penalty is applied to DD edges as opposed to the other types. Using a stability-based procedure to determine these parameters could enable more accurate predictions in practice. However, these experiments were to show impact of the parameter itself [23]. More detailed accuracy results are available, as $F_{1}$ Scores for the best performing parameter setting for each algorithm for 50 node networks can be found in Online Resource 3 (1000 Samples) and Online Resource 4 (200 Samples).

**Fig. 6:**
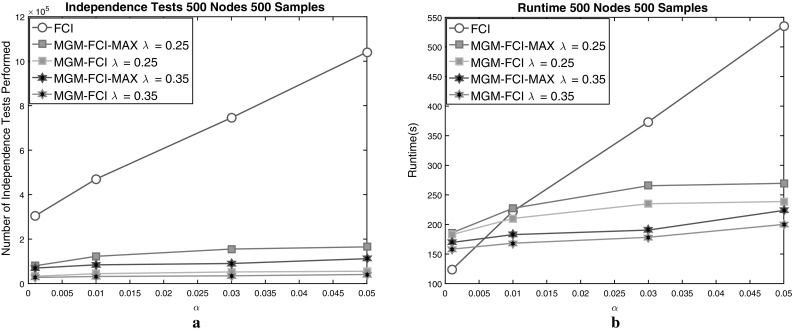
Runtime experiment with 500 nodes, 500 samples, and 20 latent variables. **a** Number of independence tests performed, **b** wall clock time

**Fig. 7:**
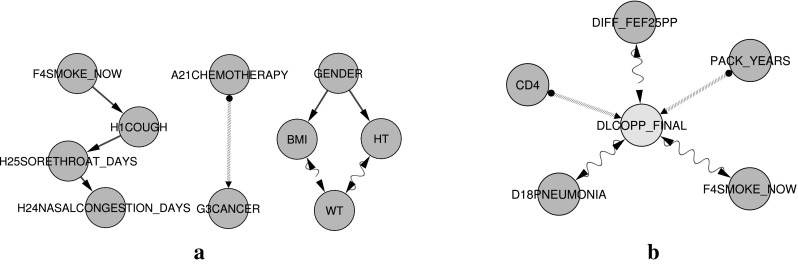
MGM-FCI-MAX predicted graphs (Lung HIV dataset). **a** Validation of output, **b** lung function variable and potential latent confounding

### Scaling to 500 node networks

To determine the scalability of the approaches, we next tested the algorithms on 500 node networks. The algorithms tested in this section were FCI, MGM-FCI, and MGM-FCI-MAX. The CFCI algorithms were not tested in this section as they did not complete a search on the 500 node networks (ran out of memory), and FCI-MAX was not tested as it had nearly identical performance to MGM-FCI-MAX but less efficient runtime. BCCD was unable to complete a search on a 500 node network within a half day so it was also excluded from this section.

Figure 5 displays the accuracy results on these networks, and a similar pattern to the smaller networks appears. The algorithms all produce similar adjacencies though MGM allows the algorithms to maintain high precision across all parameter values. In orientation accuracy, clearly MGM-FCI-MAX produces the most accurate orientations, achieving the best scores in both precision and recall with almost no effect from changing the parameters of the algorithm. FCI demonstrates a slight edge in recall of DD edges; however, the parameter setting of FCI which achieves the same precision as the MGM approaches tends to have the same recall, implying that this is a simply a precision-recall tradeoff. For more detailed results, Online Resource 5 has the $F_{1}$ scores for the best performing parameters for each algorithm.

Figure 6a, b displays the results of measuring the runtime and number of independence tests performed for these algorithms on 8 core machines. Here, we find that for larger values of the $\alpha$ independence test threshold FCI significantly slows down in comparison to the MGM-based approaches. For very small values of $\alpha$ we find no significant difference between FCI and the MGM-based approaches in run time. However, in practice, $\alpha = .05$ tends to be a commonly accepted parameter setting which results in very large runtimes using FCI. In all cases, FCI performs significantly more independence tests, and thus will certainly not be scalable if a more computationally costly independence test is used such as a Nonlinear regression test. Overall, we find that MGM-FCI-MAX tends to balance accuracy and efficiency most effectively in our simulated experiments.

### Lung HIV dataset results

Though synthetic data provide a good testing ground for algorithm evaluation, it is still constrained by the assumptions used to generate the data. Thus, in this section we examine whether MGM-FCI-MAX can identify reasonable connections on real observational data. In Figure 7, we present the results of applying MGM-FCI-MAX to the Lung HIV dataset. Figure 7a displays a section of the full causal network containing expected relationships between variables for validation of the method. Gender and weight are causally associated with both Body Mass Index (BMI) and Height, though the relationship between height and weight is mediated by a latent variable. This latent could easily be explained by underlying genetic mechanisms controlling body size. The latent variable prediction between weight and BMI may have to do with the high correlation of BMI, Height, and Weight which can pose problems to graphical models. Despite this orientation, all of these associations are expected and are common knowledge. The chemotherapy variable is causally linked to cancer, since chemotherapy remains to be a common treatment for cancer patients. The final segment of the network is further validation as smoking, coughing, sore throat, and nasal congestion are causally related.

Figure 7b displays the variables causally related to the Carbon Monoxide Diffusion Capacity percent predicted (DLCOPP) variable, which is a key target variable in this dataset as it quantifies lung function. Many of these variables have obvious direct relationships to lung function: whether or not the patient smokes (F4SMOKE NOW), the amount the patient smokes (PACK YEARS), and whether the patient has Pneumonia (D18PNEUMONIA). The DIFF FEF25PP variable corresponds to the change in an individual’s lung function test called Forced Expiratory Flow before and after bronchodilator administration. According to the PAG, these two variables are mediated by a latent confounder which makes sense given that they are two different measures of lung function, and though they should be correlated, they should not be a cause-effect relation to one another. The final linked variable is CD4 cell count which is a measure of progression of the HIV illness, or equivalently a measure of immune system decline. Recent studies have demonstrated a relationship between low CD4 count and lung illnesses including lung cancer [6] and Chronic Obstructive Pulmonary Disease (COPD) [20]. Our analysis provides evidence that the relationship may not just be a correlation, but may indeed be causally linked, warranting further study. Overall, this dataset provides a demonstration of the predictive power of MGM-FCI-MAX on real, causally insufficient, mixed datasets. The full learned causal graph and description of all variables are included in Online Resource 6 and 7, respectively.

## Conclusions

We have presented several new approaches (MGM-based approaches and FCI-MAX) and a parallelization procedure that improves the scalability of causal learning from mixed data with latent variables. The new strategies included the use of a new conditional independence test for mixed data and these were compared to the existing baseline methods (FCI-Stable, CFCI) and to another algorithm for directed graph inference over mixed data (BCCD). The comparisons included adjacency and orientation accuracy as well as run time measurements. Our results show that using the MAX search strategy to orient edges helps balance precision and recall among all edge types (CC, CD, DD). In addition, we find that using MGM for the skeleton identification overcomes the increase runtime of MAX and allows for scalability beyond FCI while maintaining the improved orientation predictions of the MAX strategy. Though determining the specific parameters to use for the algorithm is important, we note that in most cases the accuracies tend to be very similar across a wide range of parameter values which demonstrates the usability of the algorithms. We leave to future work a more detailed analysis of the limits of scalability of the MGM-based methods across parameter settings.

In addition, here we assumed linear and logistic regressions were accurate models of the interactions between continuous and discrete variables. However, it is not clear how well these assumptions will hold in certain continuous nonlinear cases. For future work, we plan to generalize our approaches to conditions where linearity is not a suitable assumption for the continuous variables. Finally, there is a pressing need to examine which of the many proposed algorithms for causal discovery are most suitable for particular graphical structures, particular distributional assumptions, and particular output metrics of interest (Structural Hamming Distance, Orientation Accuracy, etc.). Though analyses of this type were included in this work, we leave to future work a larger scale investigation of the algorithms proposed here and elsewhere under a larger variety of assumptions.

## Electronic supplementary material

Below is the link to the electronic supplementary material.

Supplementary material 1 (pdf 116 KB)

Supplementary material 2 (pdf 122 KB)

Supplementary material 3 (xlsx 9 KB)

Supplementary material 4 (xlsx 9 KB)

Supplementary material 5 (xlsx 8 KB)

Supplementary material 6 (pdf 22 KB)

Supplementary material 7 (xlsx 23 KB)
