# Supplementary material for: Comparison of strategies for scalable causal discovery of latent variable models from mixed data
Source: Int J Data Sci Anal. 2018 Feb 6;6(1):33–45. doi: 10.1007/s41060-018-0104-3 (PMC6096780; doi:10.1007/s41060-018-0104-3)
Supplement: Supplementary file 1 — Supplementary material 1 (pdf 116 KB) [file 41060_2018_104_MOESM1_ESM.pdf]

International Journal of Data Science and Analytics manuscript No.
(will be inserted by the editor)

Comparison of Strategies for Scalable Causal Discovery of Latent Variable Models from Mixed Data

Vineet K Raghu[1] · Joseph D. Ramsey[3] · Alison Morris[4] · Dimitrios V. Manatakis[2] · Peter Sprites[3] · Panos K. Chrysanthis[1] · Clark Glymour[3] · Panayiotis V. Benos[1,2]

Received: date / Accepted: date

1 Supplementary Material

The standard terminology and assumptions of this work (see, also [3]) are as follows. A graph $G = (V, E)$ consists of a set of nodes V (variables) and a set of edges E between them. A directed path is a sequence of nodes $(X_1,\ldots,X_n)$ in which there exists a directed edge from each node to the next, and where $n > 1$. A directed acyclic graph (DAG) is a graph in which no node has a directed path into itself. A DAG is a causal DAG for a population when there is a directed edge ($A \rightarrow B$) iff A is a direct cause of B in the population (relative to the variables in G). Let $Descendants(A, G)$ consist of all nodes X that have a directed path from A to X in G, and let $Ancestors(A, G)$ consist of all nodes X in which there exists a directed path from X to A in G. Finally, let $Parents(A, G)$ consist of all nodes X in which there exists a directed edge from X into A in G ($X \rightarrow A$). We omit G in the previous notations if it is clear which graph is being referred to.

Another typical assumption is that of causal sufficiency, which states that all variables that have at least two observed children in the dataset are observed themself. In our case, we do not employ this assumption as our algorithms are equipped to handle latent variables.

In order to connect causality with observational data, two assumptions are typically employed: the Causal Markov and the Causal Faithfulness assumption [1].

Vineet Raghu
E-mail: vineet@cs.pitt.edu

[1] Department of Computer Science, University of Pittsburgh ·
[2] Department of Computational and Systems Biology, University of Pittsburgh ·
[3] Department of Philosophy, Carnegie Mellon University ·
[4] UPMC Department of Medicine

Definition 1 *In a population with a causally sufficient set of variables V, a distribution $P(V)$ and a causal DAG $G = (V, E)$, $P(V)$ and G satisfy the* ***Causal Markov Assumption*** *only if*
$\forall X \in V$
$X \perp\!\!\!\perp V/(Descendants(X, G), X)|Parents(X, G)$

In a causally sufficient set of variables V, the Causal Markov assumption states that for a causal DAG G, every node of G is independent of its non descendants given its parents.

Definition 2 *In a population with a causally sufficient set of variables V, a distribution $P(V)$ and a causal DAG $G = (V, E)$ that represents the causal system in the population, $P(V)$ and G satisfy the* ***Causal Faithfulness Assumption*** *only if $X \perp\!\!\!\perp Y|Z$ in $P(V)$ only if X is d-separated from Y conditional on Z in G.*

A distribution P generated by a DAG G is *faithful* to G iff the only conditional independencies true in P are those entailed by the Causal Markov assumption applied to G. These two assumptions together are necessary to allow for reliable causal inference from observational data.

A triple is a set of three nodes (X,Y,Z) where X is adjacent to Y and Y is adjacent to Z. If X is not adjacent to Z, then this set of vertices is referred to as unshielded. If both of these edges are directed into Y, then the triple is referred to as a collider. A separating set for A and B in a causal DAG G is any set of variables S such that $A \perp\!\!\!\perp B|S \in G$.

Using these assumptions, one can define *Markov Equivalent Graphs*, which are graphs that contain the same adjacencies (same edges ignoring causal direction) and the same unshielded colliders. These graphs are equivalent in the sense that they entail exactly the same set of conditional independencies, and thus using conditional independence information alone there is no

statistical reason to favor any Markov Equivalent graph over another. When searching over datasets ignoring latent variables, causal search algorithms typically output a Partially Directed Acyclic Graph (PDAG), which represents a Markov Equivalence class of graphs by representing edges with the same orientation by all graphs in an equivalence class with a directed edge, and representing all other edges in the equivalence class by an undirected edge.

Without causal sufficiency, PDAGs are no longer sufficient to represent the complete and correct independence information from the observed variables, so instead ancestral graphical models are used [2]. In particular, Maximal Ancestral Graphs (MAGs) are causal graphical structures that can include directed edges ($\rightarrow$) as well as bi-directed edges ($\leftrightarrow$), where the bi-directed edges are indicative of a latent common cause between the variables participating in the edge. MAGs may also include undirected edges indicative of selection bias; however in this work selection bias is ignored. Intuitively, a MAG provides all of the conditional independence information that can be derived from a DAG where only a subset of the variables are measured. In particular, adjacencies in a MAG are defined as follows:

1. There is an adjacency between A and B in a MAG for a DAG G when A and B are d-connected conditional on every subset of the observed variables.

2. There is an arrowhead on an edge ($A \rightarrow B or A \leftrightarrow B$) when B is not an ancestor of A and a tail on an edge ($A \rightarrow B$) when A is an ancestor of B.

As is the case with DAGs, multiple MAGs can entail the same Markov independence relations, and we refer to these MAGs as an equivalence class. Thus the goal of the algorithms in this work is to provide this equivalence class upon which independence relations, and subsequently, causal relations can be determined. An equivalence class of MAGs is referred to as a Partial Ancestral Graph (PAG). A PAG portrays the information contained in a class of MAGs using the following three conditions: [4]

1. The PAG has the same adjacencies as every MAG in the equivalence class.
2. If the endpoint of an edge in every MAG in the class is an arrowhead or a tail, then the endpoint of this edge in the PAG will be an arrowhead or a tail respectively.
3. For all edge endpoints that are not the same in every MAG, the PAG uses the mark of (o) to denote this.

Thus, an edge in the PAG of the form A o$\rightarrow$ B implies only that B is not an ancestor of A, and no information about the ancestral relationship from A to B is implied.

References

1. Pearl, J.: Probabilistic Reasoning in Intelligent Systems (1988). DOI 10.2307/2026705
2. Richardson, T., Spirtes, P.: Ancestral graph Markov models. Annals of Statistics **30**(4), 962–1030 (2002). DOI 10.1214/aos/1031689015
3. Spirtes, P., Glymour, C., Scheines, R.: Causation, Prediction, and Search. Technometrics **45**(3), 272–273 (2003). DOI 10.1198/tech.2003.s776
4. Zhang, J.: On the completeness of orientation rules for causal discovery in the presence of latent confounders and selection bias. Artificial Intelligence **172**(16-17), 1873–1896 (2008). DOI 10.1016/j.artint.2008.08.001

Table 1: Graph Statistics for 50 Node Networks

Graph ID	Number of Edges	Max Node Degree	Average Node Degree	Standard Deviation Node Degrees
0	68	6.0	2.72	1.3099306727254456
1	48	4.0	1.92	1.1752550743544647
2	101	7.0	4.04	1.8065639953756842
3	112	10.0	4.48	2.2336937469362357
4	54	7.0	2.16	1.4757910365879952
5	64	7.0	2.56	1.6182631152578622
6	123	10.0	4.92	2.310932314730422
7	79	6.0	3.16	1.516709650284228
8	150	10.0	6.0	2.3211538298959886
9	73	7.0	2.92	1.7709676819784843

Table 2: Graph Statistics for 500 Node Networks

Graph ID	Number of Edges	Max Node Degree	Average Node Degree	Standard Deviation Node Degrees
0	673	10.0	2.692	1.6353852665166908
1	967	14.0	3.868	2.1651678696599035
2	848	9.0	3.392	1.8365952641582883
3	796	9.0	3.184	1.781148218974464
4	731	8.0	2.924	1.6197132282857265
