# Supplementary figures and images for: Comparison of strategies for scalable causal discovery of latent variable models from mixed data
Source: Int J Data Sci Anal. 2018 Feb 6;6(1):33–45. doi: 10.1007/s41060-018-0104-3 (PMC6096780; doi:10.1007/s41060-018-0104-3)

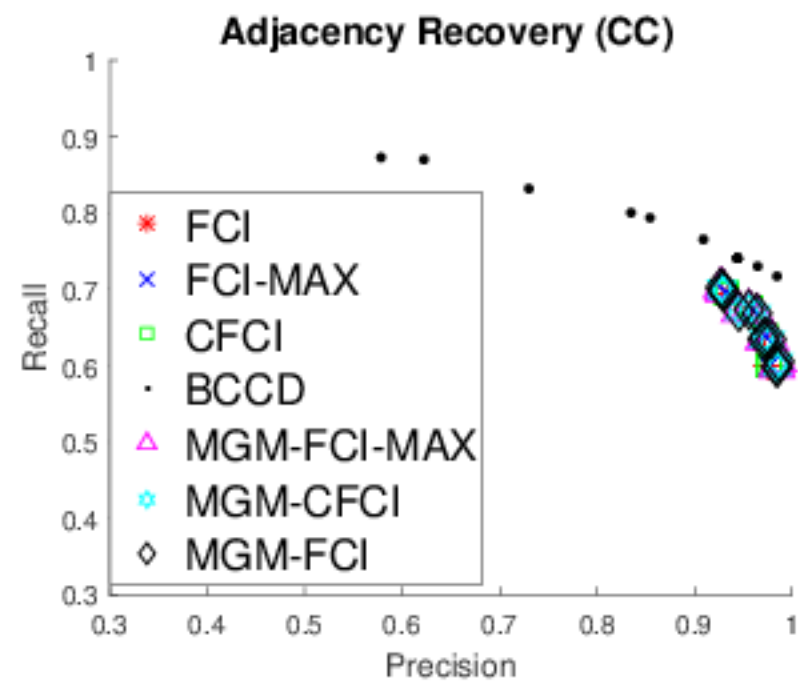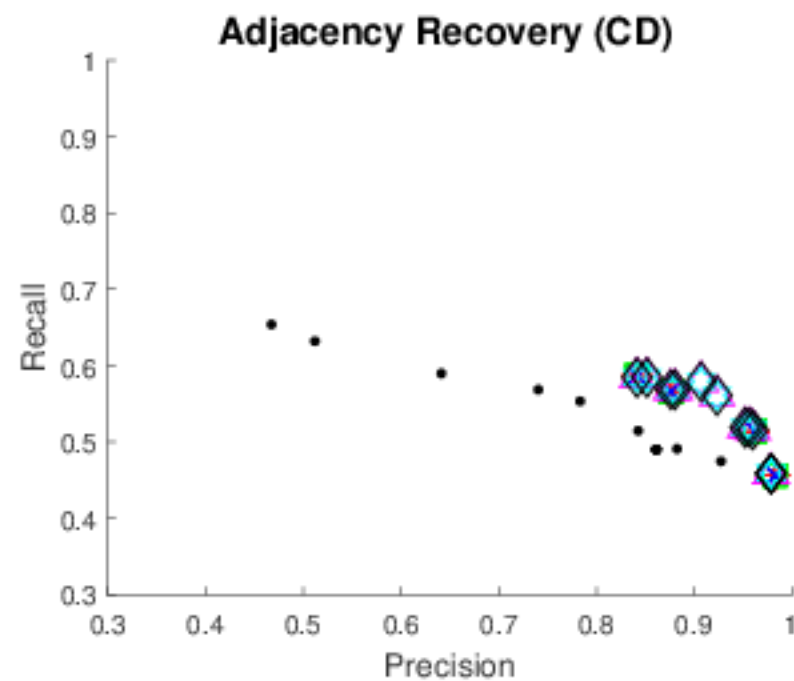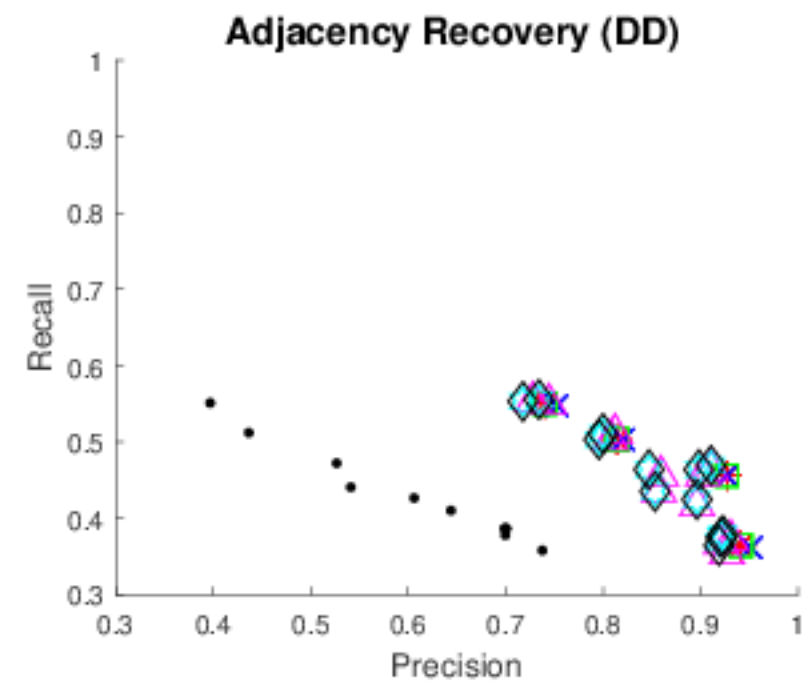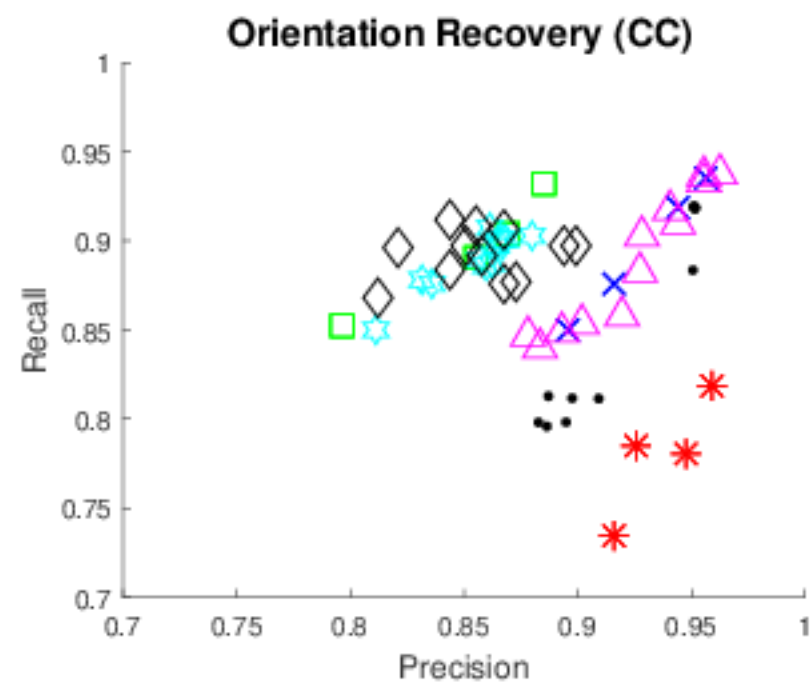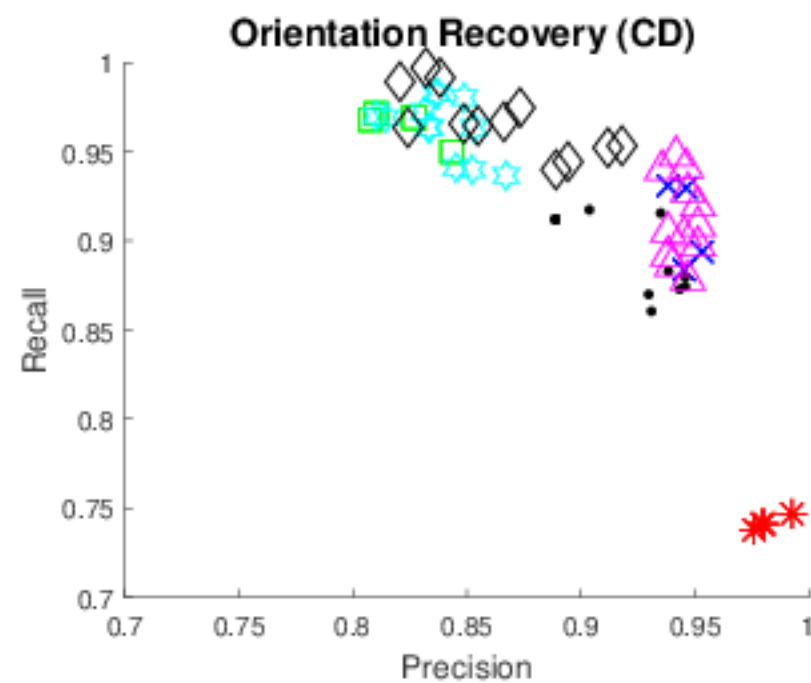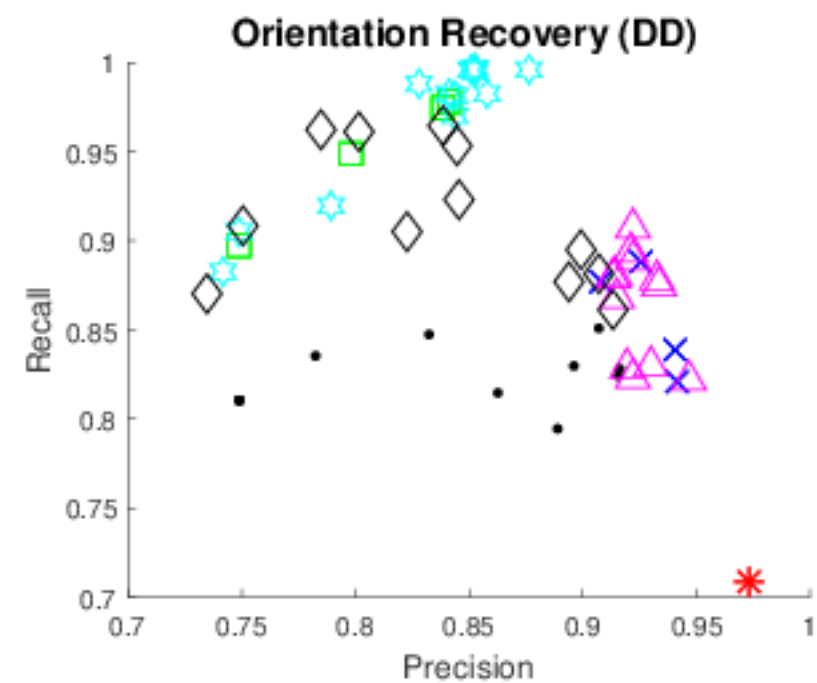

Supplement: Supplementary file 2 — Supplementary material 2 (pdf 122 KB) [file 41060_2018_104_MOESM2_ESM.pdf]

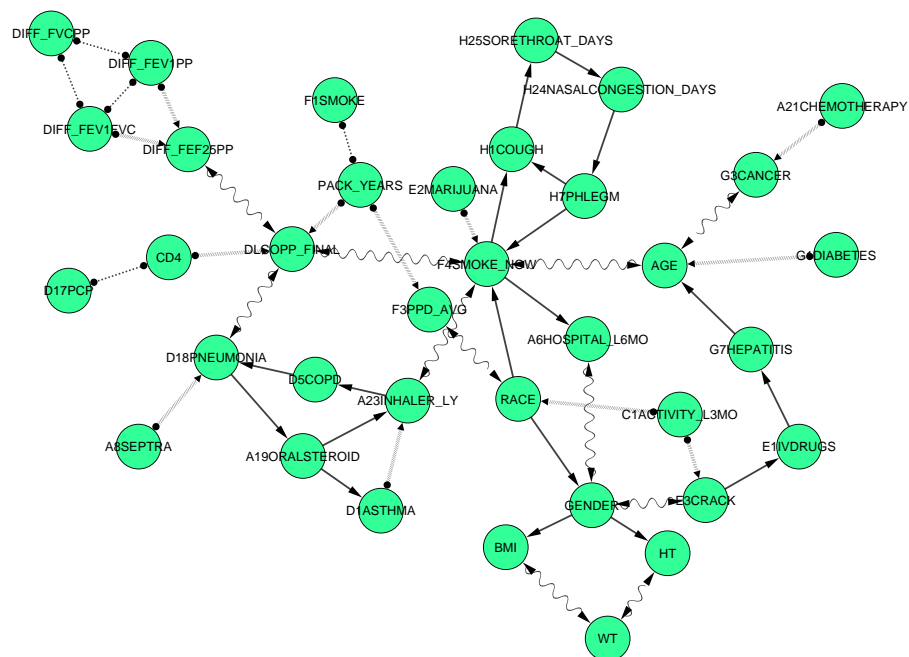

Supplement: Supplementary file 6 — Supplementary material 6 (pdf 22 KB) [file 41060_2018_104_MOESM6_ESM.pdf]
